# Navigating the Diagnostic Journey: Insights from Frontotemporal Dementia Caregivers

**DOI:** 10.1002/alz70858_107661

**Published:** 2025-12-26

**Authors:** Grace Mausisa, Annick de Bruin, Devon M Chenette, Susan Gorky, Tiffany W Chow

**Affiliations:** ^1^ Alector, South San Francisco, CA, USA; ^2^ Center for Information and Study on Clinical Research Participation, Boston, MA, USA

## Abstract

**Background:**

Caregivers play critical roles in supporting those diagnosed with frontotemporal dementia (FTD). We surveyed caregivers to gain a deeper understanding of their experience with the diagnostic journey and the clinical support received from healthcare professionals.

**Method:**

Alector developed the FTD Caregiver Survey, an online self‐administered questionnaire. The survey was distributed with support from patient groups, individual advocates and an FTD website community. Informed consent was collected. Eligible responders were adults who could read and write in English, reside in the United States, and who have been an unpaid primary caregiver for a person diagnosed with FTD.

**Result:**

Of 106 respondents, 58% sought medical help within 1 year, 15% between 1‐2 years, and 27% sought medical help more than 2 years after first noticing symptoms. Of those who reported waiting > 1 year after noticing symptoms (*n* = 45), 64% had been unaware of FTD and 49% thought the symptoms were part of normal life changes. A larger percentage of rural respondents waited > 2 years compared to suburban respondents (50% vs. 19%, nominal *p* <0.05; too few respondents from urban areas to be compared statistically).

Lack of knowledge about FTD among healthcare professionals visited (54%) and misdiagnosis (42%) were most frequently identified as challenges to receiving a diagnosis. Respondents acknowledged receiving symptom management (58%), learning about caregiver support (32%) and educational resources (26%) from their providers. Thirty‐three percent reported that they had not discussed any of these topics with their providers. Thirty‐six percent were informed about clinical research. When asked which providers were most needed, caregivers selected neurologists with FTD experience (76%), primary care providers (42%), and case managers/social workers (41%) as the top healthcare professionals. Neurologists with experience in FTD were the most difficult to find and access (58%).

**Conclusion:**

It is critical to understand the perspectives and challenges of caregivers who play a primary role in the diagnosis and care of individuals with FTD. These findings highlight the importance of raising public awareness regarding early symptoms of FTD. Continued FTD education efforts among healthcare professionals may also help to advance the early and accurate diagnosis of FTD and increase the support provided to caregivers.

